# 2,9-Di-3-pentyl­anthra[1,9-*def*:6,5,10-*d*′*e*′*f*′]diisoquinoline-1,3,8,10-tetrone

**DOI:** 10.1107/S1600536810036275

**Published:** 2010-09-15

**Authors:** Waldemar Maniukiewicz, Joanna Bojarska, Andrzej Olczak, Ewa Dobruchowska, Michał Wiatrowski

**Affiliations:** a Institute of General and Ecological Chemistry, Technical University of Łódź, Żeromskiego 116, 90-924 Łódź, Poland; b Department of Molecular Physics, Technical University of Łódź, Żeromskiego 116, 90-924 Łódź, Poland

## Abstract

The asymmetric unit of the title compound, C_34_H_30_N_2_O_4_, contains four independent half-mol­ecules, the complete mol­ecules being generated by inversion symmetry. The mol­ecules each have planar (within 4σ) perylene­tetra­carb­oxy­lic diimide fragments with bent side chains. In one of the independent mol­ecules, each 3-pentyl fragment is disordered over two conformations in a 7:3 ratio. In the crystal, π–π inter­actions link mol­ecules into stacks propagated in [010]. The crystal packing also exhibits weak inter­molecular C—H⋯O hydrogen bonds.

## Related literature

For the properties and applications of perylene derivatives, see: Tracz *et al.* (1981 ▶); Graser & Hädicke (1980 ▶, 1984 ▶); Herbst & Hunger (1993 ▶); Newman *et al.* (2004 ▶); Wurthner (2004 ▶); Dodabalapur (2006 ▶); Miśkiewicz *et al.* (2006 ▶); Wiatrowski *et al.* (2010 ▶). For related structures, see: Hädicke & Graser (1986*a* ▶,*b* ▶); Mizuguchi (2003 ▶); Guillermet *et al.* (2006 ▶); Briseno *et al.* (2007 ▶).
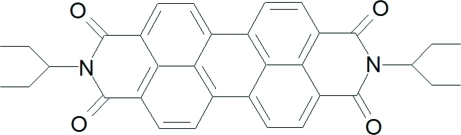


## Experimental

### 

#### Crystal data

C_34_H_30_N_2_O_4_
*M* *_r_* = 530.60
Monoclinic, 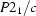

*a* = 34.009 (4) Å
*b* = 7.5035 (10) Å
*c* = 21.248 (3) Å
β = 100.562 (2)°
*V* = 5330.3 (12) Å^3^
*Z* = 8
Mo *K*α radiation
μ = 0.09 mm^−1^
*T* = 296 K
0.40 × 0.05 × 0.05 mm

#### Data collection

Bruker SMART APEXII CCD diffractometer
Absorption correction: multi-scan (*SADABS*; Sheldrick, 2002 ▶) *T*_min_ = 0.966, *T*_max_ = 0.996
85917 measured reflections
9351 independent reflections
6350 reflections with *I* > 2σ(*I*)
*R*_int_ = 0.051

#### Refinement

*R*[*F* ^2^ > 2σ(*F* ^2^)] = 0.062
*wR*(*F* ^2^) = 0.166
*S* = 1.03
9351 reflections
760 parameters
871 restraints
H-atom parameters constrained
Δρ_max_ = 0.24 e Å^−3^
Δρ_min_ = −0.21 e Å^−3^

### 

Data collection: *SMART* (Bruker, 2003 ▶); cell refinement: *SAINT* (Bruker, 2003 ▶); data reduction: *SAINT*; program(s) used to solve structure: *SHELXS97* (Sheldrick, 2008 ▶); program(s) used to refine structure: *SHELXL97* (Sheldrick, 2008 ▶); molecular graphics: *ORTEP-3* (Farrugia, 1997 ▶) and *PLATON* (Spek, 2009 ▶); software used to prepare material for publication: *publCIF* (Westrip, 2010 ▶).

## Supplementary Material

Crystal structure: contains datablocks I, global. DOI: 10.1107/S1600536810036275/cv2759sup1.cif

Structure factors: contains datablocks I. DOI: 10.1107/S1600536810036275/cv2759Isup2.hkl

Additional supplementary materials:  crystallographic information; 3D view; checkCIF report

## Figures and Tables

**Table 1:** Centroid–centroid distances (Å) *Cg*1, *Cg*2, *Cg*3, *Cg*4, *Cg*5, *Cg*6, *Cg*7, *Cg*8, *Cg*9, *Cg*10, *Cg*11, *Cg*12, *Cg*13 and *Cg*14 are the centroids of the N4/C52–C56, C53/C54/C57–C60, C54/C55/C60–C63, C59–C61/C59^*d*^–C61^*d*^, C2/C3/C6–C9, C3/C4/C9–C12, C8–C10/C8^*a*^–C10^*a*^, N2/C18–C22, C19/C20/C23–C26, C25–C27/C25^*b*^–C27^*b*^, N3/C35–C39, C36/C37/C40–C43, C37/C38/C43–C46 and C42–C44/C42^*c*^–C44^*c*^, respectively. Symmetry codes: (*a*) 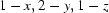; (*b*) 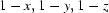; (*c*) 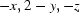; (*d*) 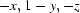.

*Cg*1⋯*Cg*13^i^	3.6863 (19)
*Cg*2⋯*Cg*11	3.7041 (19)
*Cg*2⋯*Cg*12^i^	3.6485 (18)
*Cg*2⋯*Cg*13^i^	3.6165 (18)
*Cg*3⋯*Cg*13	3.7245 (19)
*Cg*4⋯*Cg*13	3.6034 (18)
*Cg*4⋯*Cg*14^i^	3.7518 (18)
*Cg*5⋯*Cg*9^ii^	3.6604 (16)
*Cg*6⋯*Cg*10	3.5395 (15)
*Cg*6⋯*Cg*8^ii^	3.7743 (16)
*Cg*6⋯*Cg*9	3.7544 (16)
*Cg*7⋯*Cg*9^ii^	3.5613 (15)
*Cg*7⋯*Cg*10	3.7518 (14)

Symmetry codes: (i) 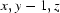; (ii) 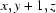.

**Table 2:** Hydrogen-bond geometry (Å, °)

*D*—H⋯*A*	*D*—H	H⋯*A*	*D*⋯*A*	*D*—H⋯*A*
C7—H7⋯O4^iii^	0.93	2.55	3.170 (4)	125
C15—H15*B*⋯O3^ii^	0.96	2.42	3.323 (5)	156
C32—H32*C*⋯O2	0.96	2.60	3.453 (5)	148
C41—H41⋯O8^iv^	0.93	2.49	3.343 (4)	153
C49—H49*C*⋯O7^ii^	0.96	2.56	3.481 (6)	162

Symmetry codes: (ii) 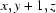; (iii) 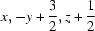; (iv) 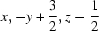.

**Fig. 1.**
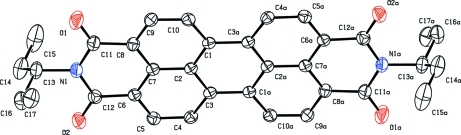
One independent molecule of the title compound showing the atomic numbering and 30% probability displacement ellipsoids [symmetry code: (a) -x, 1 - y, -z]. All H atoms have been omitted for clarity.

**Fig. 2.**
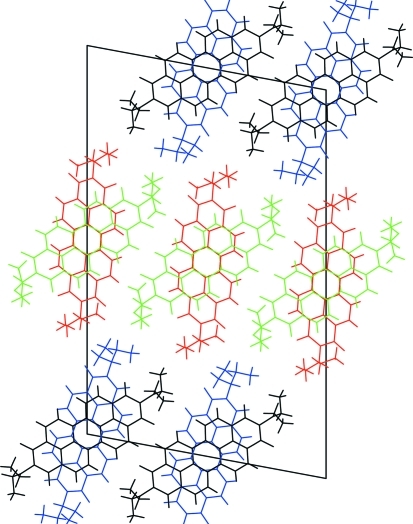
Molecular packing viewed down the b.

## supplementary crystallographic information

### Comment

The derivatives of perylene tetracarboxylic diimide (PTCDI) have long been a focus of research for their wide applications. These compounds are well known as industrially important organic pigments, which exhibit a variety of colours (Herbst & Hunger, 1993). The correlations between the colour of the crystals and the different arrangement of the molecules in the stacks were discussed in details by Graser and Hädicke (Graser and Hädicke,1980, 1984). Besides, the derivatives of perylenediimide are extremely robust, commercially available materials with high photo and thermal stability (Newman, 2004). More recently the PTCDI's are also of interest as the n-type semiconductors, in contrast to the more common *p*-type counterparts in organic semiconductors, making them attractive candidates for fabrication of various organic electronic elements (Wiatrowski *et al.*, 2010; Dodabalapur, 2006; Miśkiewicz, 2006; Wurthner, 2004; Tracz, 1981). The cystal and molecular structure of the title compound reported here have been determined as part of an investigation on the charge transport properties of organic semiconductors caused by electronic coupling between stacking molecules. The unit structure of the PTCDI is illustrated in Fig. 1. Neighbouring molecules are arranged in stacks (Fig. 2), where the stacking distance is about 3.35 Å, with their long axes tilted in relation to the column grow direction. Typical hydrogen bonds are not observed.

### Experimental

All chemicals used were commercially available. PTCDI-C5(3) was provided SynTech GmbH. The single crystals were grown by slow evaporation from a nitrobenzene solution of the title compound at room temperature. After several weeks poor quality, weakly diffracted dark red needle crystals were obtained. The structure was determined by single-crystal diffraction.

### Refinement

All hydrogen atoms were placed in geometrically idealized positions and constrained to ride on their parent atoms, with C—H = 0.93 - 0.97 Å and with U_iso_(H) = 1.2-1.5 U_eq_(C). The terminal ethyl groups in one of the molecules were treated as disordered over two sets of positions using restraints on the anisotropic displacement, with the occupancies fixed to 0.7 and 0.3, respectively.

### Crystal data

**Table:**

C_34_H_30_N_2_O_4_	*F*(000) = 2240
*M**_r_* = 530.60	*D*_x_ = 1.322 Mg m^−^^3^
Monoclinic, *P*2_1_/*c*	Mo *K*α radiation, λ = 0.71073 Å
Hall symbol: -P 2ybc	Cell parameters from 9930 reflections
*a* = 34.009 (4) Å	θ = 2.4–26.2°
*b* = 7.5035 (10) Å	µ = 0.09 mm^−^^1^
*c* = 21.248 (3) Å	*T* = 296 K
β = 100.562 (2)°	Needle, dark red
*V* = 5330.3 (12) Å^3^	0.40 × 0.05 × 0.05 mm
*Z* = 8	

### Data collection

**Table:**

Bruker APEXII CCD diffractometer	9351 independent reflections
Radiation source: fine-focus sealed tube	6350 reflections with *I* > 2σ(*I*)
graphite	*R*_int_ = 0.051
ω scan	θ_max_ = 25.0°, θ_min_ = 1.8°
Absorption correction: multi-scan (*SADABS*; Sheldrick, 2002)	*h* = −40→40
*T*_min_ = 0.966, *T*_max_ = 0.996	*k* = −8→8
85917 measured reflections	*l* = −25→25

### Refinement

**Table:**

Refinement on *F*^2^	Primary atom site location: structure-invariant direct methods
Least-squares matrix: full	Secondary atom site location: difference Fourier map
*R*[*F*^2^ > 2σ(*F*^2^)] = 0.062	Hydrogen site location: inferred from neighbouring sites
*wR*(*F*^2^) = 0.166	H-atom parameters constrained
*S* = 1.02	*w* = 1/[σ^2^(*F*_o_^2^) + (0.0469*P*)^2^ + 6.5326*P*] where *P* = (*F*_o_^2^ + 2*F*_c_^2^)/3
9351 reflections	(Δ/σ)_max_ < 0.001
760 parameters	Δρ_max_ = 0.24 e Å^−^^3^
871 restraints	Δρ_min_ = −0.21 e Å^−^^3^

### Special details

**Table:**

Geometry. Bond distances, angles *etc*. have been calculated using the rounded fractional coordinates. All su's are estimated from the variances of the (full) variance-covariance matrix. The cell e.s.d.'s are taken into account in the estimation of distances, angles and torsion angles
Refinement. Refinement of *F*^2^ against ALL reflections. The weighted *R*-factor *wR* and goodness of fit *S* are based on *F*^2^, conventional *R*-factors *R* are based on *F*, with *F* set to zero for negative *F*^2^. The threshold expression of *F*^2^ > σ(*F*^2^) is used only for calculating *R*-factors(gt) *etc*. and is not relevant to the choice of reflections for refinement. *R*-factors based on *F*^2^ are statistically about twice as large as those based on *F*, and *R*- factors based on ALL data will be even larger.

### Fractional atomic coordinates and isotropic or equivalent isotropic displacement parameters (Å^2^)

**Table:**

	*x*	*y*	*z*	*U*_iso_*/*U*_eq_	Occ. (<1)
O7	0.15032 (8)	0.1736 (4)	0.15767 (13)	0.0912 (11)	
O8	0.05685 (9)	0.2928 (5)	0.27973 (12)	0.1039 (13)	
N4	0.10523 (9)	0.2440 (4)	0.22107 (14)	0.0698 (10)	
C52	0.11766 (11)	0.2356 (5)	0.16172 (17)	0.0672 (12)	
C53	0.09065 (10)	0.3063 (4)	0.10547 (16)	0.0593 (11)	
C54	0.05202 (9)	0.3650 (4)	0.11002 (15)	0.0543 (10)	
C55	0.03954 (11)	0.3581 (5)	0.16981 (16)	0.0641 (11)	
C56	0.06682 (12)	0.2964 (5)	0.22741 (18)	0.0745 (14)	
C57	0.10337 (10)	0.3144 (4)	0.04753 (17)	0.0622 (11)	
C58	0.07854 (10)	0.3848 (4)	−0.00564 (16)	0.0607 (11)	
C59	0.03994 (9)	0.4419 (4)	−0.00431 (14)	0.0518 (10)	
C60	0.02605 (9)	0.4311 (4)	0.05535 (14)	0.0508 (10)	
C61	−0.01339 (9)	0.4882 (4)	0.06022 (15)	0.0546 (11)	
C62	−0.02436 (11)	0.4748 (5)	0.12051 (16)	0.0675 (11)	
C63	0.00152 (11)	0.4127 (5)	0.17361 (17)	0.0743 (14)	
C64A	0.1356 (3)	0.1883 (11)	0.2766 (4)	0.081 (3)	0.700
C65A	0.1658 (3)	0.3118 (13)	0.2996 (5)	0.179 (5)	0.700
C66A	0.1864 (3)	0.3551 (14)	0.3593 (4)	0.180 (5)	0.700
C67A	0.1251 (3)	0.0055 (14)	0.3020 (4)	0.128 (4)	0.700
C68A	0.1542 (3)	−0.0819 (14)	0.3474 (4)	0.142 (4)	0.700
C64B	0.1310 (8)	0.204 (2)	0.2901 (12)	0.095 (5)	0.300
C65B	0.1457 (4)	0.3684 (15)	0.3128 (5)	0.132 (6)	0.300
C66B	0.1875 (4)	0.4319 (15)	0.3069 (5)	0.188 (9)	0.300
C67B	0.1426 (4)	0.0054 (15)	0.2839 (5)	0.087 (6)	0.300
C68B	0.1231 (8)	−0.110 (3)	0.3206 (12)	0.158 (13)	0.300
O1	0.33801 (6)	0.8756 (4)	0.57655 (11)	0.0825 (9)	
O2	0.34231 (6)	0.6514 (3)	0.37896 (10)	0.0623 (8)	
N1	0.33851 (6)	0.7642 (3)	0.47692 (11)	0.0493 (8)	
C1	0.35654 (8)	0.8479 (4)	0.53397 (14)	0.0531 (10)	
C2	0.39920 (8)	0.8984 (4)	0.54060 (12)	0.0420 (8)	
C3	0.41964 (7)	0.8790 (3)	0.48919 (12)	0.0365 (8)	
C4	0.39983 (7)	0.8014 (3)	0.43157 (12)	0.0384 (8)	
C5	0.35849 (8)	0.7328 (4)	0.42623 (14)	0.0469 (9)	
C6	0.41887 (8)	0.9708 (4)	0.59704 (13)	0.0467 (9)	
C7	0.45877 (8)	1.0237 (4)	0.60401 (12)	0.0433 (9)	
C8	0.47981 (7)	1.0109 (3)	0.55426 (11)	0.0353 (8)	
C9	0.45979 (7)	0.9384 (3)	0.49465 (11)	0.0340 (8)	
C10	0.47885 (7)	0.9254 (3)	0.44059 (11)	0.0356 (8)	
C11	0.45780 (8)	0.8520 (4)	0.38439 (12)	0.0413 (8)	
C12	0.41902 (8)	0.7882 (4)	0.38058 (12)	0.0420 (8)	
C13	0.29580 (8)	0.7047 (5)	0.47125 (16)	0.0606 (11)	
C14	0.26833 (10)	0.7838 (6)	0.41446 (19)	0.0830 (15)	
C15	0.27052 (12)	0.9843 (7)	0.4094 (3)	0.117 (2)	
C16	0.29299 (10)	0.5022 (5)	0.47604 (18)	0.0714 (11)	
C17	0.31410 (11)	0.4269 (6)	0.53911 (17)	0.0790 (16)	
O3	0.34920 (6)	0.1448 (3)	0.35559 (9)	0.0598 (8)	
O4	0.43654 (7)	0.2748 (4)	0.22385 (9)	0.0716 (9)	
N2	0.39047 (7)	0.2227 (3)	0.28708 (10)	0.0480 (8)	
C18	0.38009 (8)	0.2150 (4)	0.34781 (13)	0.0456 (9)	
C19	0.40806 (7)	0.2938 (3)	0.40210 (12)	0.0379 (8)	
C20	0.44575 (7)	0.3567 (3)	0.39396 (12)	0.0358 (8)	
C21	0.45633 (8)	0.3498 (4)	0.33281 (12)	0.0419 (8)	
C22	0.42768 (8)	0.2810 (4)	0.27691 (13)	0.0494 (10)	
C23	0.39707 (7)	0.3066 (4)	0.46086 (12)	0.0414 (8)	
C24	0.42302 (7)	0.3787 (4)	0.51279 (12)	0.0409 (8)	
C25	0.46077 (7)	0.4389 (3)	0.50770 (12)	0.0354 (8)	
C26	0.47294 (7)	0.4267 (3)	0.44697 (11)	0.0343 (8)	
C27	0.51111 (7)	0.4883 (3)	0.43760 (12)	0.0364 (8)	
C28	0.52010 (8)	0.4797 (4)	0.37648 (13)	0.0471 (9)	
C29	0.49306 (8)	0.4127 (4)	0.32512 (13)	0.0500 (10)	
C30	0.36052 (9)	0.1622 (4)	0.23004 (14)	0.0557 (10)	
C31	0.32038 (10)	0.2563 (5)	0.22485 (17)	0.0719 (14)	
C32	0.32279 (13)	0.4558 (6)	0.2294 (2)	0.0948 (17)	
C33	0.35716 (10)	−0.0399 (5)	0.22768 (15)	0.0641 (11)	
C34	0.39376 (11)	−0.1330 (5)	0.21307 (17)	0.0762 (16)	
O5	0.17448 (7)	0.8403 (4)	−0.00898 (13)	0.0895 (10)	
O6	0.13990 (7)	0.6532 (3)	0.17751 (11)	0.0713 (9)	
N3	0.15775 (7)	0.7338 (4)	0.08308 (13)	0.0601 (9)	
C35	0.14885 (10)	0.8186 (5)	0.02337 (17)	0.0622 (11)	
C36	0.10703 (9)	0.8746 (4)	0.00049 (15)	0.0521 (10)	
C37	0.07921 (8)	0.8713 (4)	0.04216 (13)	0.0448 (9)	
C38	0.09080 (9)	0.8051 (4)	0.10487 (14)	0.0498 (9)	
C39	0.13092 (9)	0.7255 (4)	0.12528 (15)	0.0543 (10)	
C40	0.09541 (9)	0.9339 (4)	−0.06097 (15)	0.0588 (11)	
C41	0.05654 (9)	0.9907 (4)	−0.08285 (14)	0.0531 (10)	
C42	0.02842 (8)	0.9957 (4)	−0.04315 (12)	0.0437 (9)	
C43	0.03971 (8)	0.9351 (4)	0.02129 (12)	0.0431 (8)	
C44	0.01245 (8)	0.9381 (4)	0.06526 (13)	0.0440 (9)	
C45	0.02573 (9)	0.8754 (4)	0.12668 (13)	0.0520 (10)	
C46	0.06412 (9)	0.8086 (4)	0.14628 (14)	0.0523 (10)	
C47	0.19744 (10)	0.6458 (5)	0.10204 (19)	0.0745 (14)	
C48	0.23055 (11)	0.7769 (7)	0.1231 (2)	0.101 (2)	
C49	0.22923 (14)	0.8710 (8)	0.1838 (3)	0.135 (3)	
C50	0.20487 (12)	0.5105 (6)	0.0501 (2)	0.0964 (18)	
C51	0.23083 (16)	0.3605 (7)	0.0769 (3)	0.139 (3)	
H65A	0.18670	0.28410	0.27570	0.2140*	0.700
H65B	0.15470	0.42470	0.28250	0.2140*	0.700
H66A	0.20720	0.43820	0.35540	0.2160*	0.700
H66B	0.19790	0.24930	0.38050	0.2160*	0.700
H66C	0.16840	0.40800	0.38390	0.2160*	0.700
H67A	0.10140	0.02050	0.32080	0.1530*	0.700
H67B	0.11800	−0.07340	0.26550	0.1530*	0.700
H68A	0.14410	−0.19610	0.35740	0.1710*	0.700
H68B	0.16000	−0.01130	0.38560	0.1710*	0.700
H68C	0.17810	−0.09800	0.33010	0.1710*	0.700
H57	0.12860	0.27250	0.04420	0.0750*	
H58	0.08820	0.39420	−0.04380	0.0730*	
H62	−0.05000	0.50910	0.12480	0.0810*	
H63	−0.00690	0.40760	0.21280	0.0890*	
H64A	0.15210	0.13290	0.24870	0.0980*	0.700
H64B	0.10940	0.19010	0.31460	0.1140*	0.300
H65C	0.14430	0.37190	0.35800	0.1590*	0.300
H65D	0.12710	0.45730	0.29200	0.1590*	0.300
H66D	0.19410	0.53680	0.33250	0.2250*	0.300
H66E	0.18810	0.45930	0.26290	0.2250*	0.300
H66F	0.20660	0.33980	0.32140	0.2250*	0.300
H67C	0.13590	−0.02960	0.23930	0.1050*	0.300
H67D	0.17130	−0.00730	0.29750	0.1050*	0.300
H68D	0.13430	−0.22740	0.32030	0.1870*	0.300
H68E	0.09510	−0.11420	0.30260	0.1870*	0.300
H68F	0.12670	−0.06700	0.36380	0.1870*	0.300
H6	0.40540	0.98470	0.63110	0.0560*	
H7	0.47160	1.06870	0.64320	0.0520*	
H11	0.46990	0.84530	0.34860	0.0490*	
H12	0.40600	0.73590	0.34280	0.0500*	
H13	0.28670	0.75200	0.50910	0.0730*	
H14A	0.24100	0.75070	0.41670	0.0990*	
H14B	0.27470	0.73170	0.37580	0.0990*	
H15A	0.25230	1.02370	0.37200	0.1400*	
H15B	0.29720	1.01870	0.40600	0.1400*	
H15C	0.26340	1.03770	0.44680	0.1400*	
H16A	0.26500	0.46860	0.46960	0.0860*	
H16B	0.30430	0.44900	0.44180	0.0860*	
H17A	0.31120	0.29960	0.53870	0.0950*	
H17B	0.30260	0.47620	0.57330	0.0950*	
H17C	0.34200	0.45690	0.54540	0.0950*	
H23	0.37200	0.26650	0.46600	0.0500*	
H24	0.41480	0.38670	0.55210	0.0490*	
H28	0.54490	0.51980	0.36990	0.0570*	
H29	0.49990	0.41020	0.28470	0.0600*	
H30	0.37130	0.19760	0.19220	0.0670*	
H31A	0.30380	0.22450	0.18430	0.0860*	
H31B	0.30720	0.21250	0.25860	0.0860*	
H32A	0.29630	0.50430	0.22570	0.1140*	
H32B	0.33500	0.50130	0.19540	0.1140*	
H32C	0.33850	0.48930	0.26990	0.1140*	
H33A	0.35210	−0.08210	0.26860	0.0770*	
H33B	0.33430	−0.07260	0.19530	0.0770*	
H34A	0.38950	−0.25950	0.21230	0.0910*	
H34B	0.41650	−0.10420	0.24550	0.0910*	
H34C	0.39860	−0.09450	0.17210	0.0910*	
H40	0.11380	0.93620	−0.08840	0.0710*	
H41	0.04910	1.02630	−0.12530	0.0640*	
H45	0.00840	0.87810	0.15580	0.0620*	
H46	0.07180	0.76590	0.18780	0.0630*	
H47	0.19550	0.57440	0.14000	0.0890*	
H48A	0.25590	0.71430	0.12760	0.1220*	
H48B	0.23000	0.86510	0.08960	0.1220*	
H49A	0.25080	0.95510	0.19220	0.1620*	
H49B	0.23180	0.78620	0.21810	0.1620*	
H49C	0.20420	0.93270	0.18040	0.1620*	
H50A	0.17940	0.46370	0.02840	0.1160*	
H50B	0.21720	0.57220	0.01860	0.1160*	
H51A	0.23470	0.28180	0.04290	0.1670*	
H51B	0.21840	0.29650	0.10710	0.1670*	
H51C	0.25620	0.40570	0.09800	0.1670*	

### Atomic displacement parameters (Å^2^)

**Table:**

	*U*^11^	*U*^22^	*U*^33^	*U*^12^	*U*^13^	*U*^23^
O7	0.0821 (18)	0.100 (2)	0.0935 (19)	0.0188 (16)	0.0214 (15)	0.0102 (16)
O8	0.123 (2)	0.134 (3)	0.0587 (15)	0.025 (2)	0.0270 (15)	−0.0005 (17)
N4	0.0826 (19)	0.0592 (18)	0.0664 (17)	−0.0051 (15)	0.0103 (14)	−0.0037 (14)
C52	0.078 (2)	0.052 (2)	0.073 (2)	−0.0053 (18)	0.0179 (18)	−0.0009 (17)
C53	0.067 (2)	0.0451 (18)	0.0688 (19)	−0.0071 (15)	0.0201 (16)	−0.0103 (15)
C54	0.0663 (19)	0.0416 (17)	0.0589 (18)	−0.0083 (15)	0.0215 (15)	−0.0124 (14)
C55	0.080 (2)	0.058 (2)	0.0578 (18)	−0.0048 (18)	0.0222 (16)	−0.0073 (16)
C56	0.093 (3)	0.069 (2)	0.064 (2)	−0.002 (2)	0.0209 (19)	−0.0052 (19)
C57	0.0597 (19)	0.055 (2)	0.077 (2)	−0.0027 (16)	0.0259 (17)	−0.0080 (17)
C58	0.066 (2)	0.059 (2)	0.0645 (19)	−0.0056 (17)	0.0318 (16)	−0.0084 (16)
C59	0.0623 (18)	0.0424 (17)	0.0562 (17)	−0.0101 (14)	0.0256 (15)	−0.0129 (14)
C60	0.0619 (18)	0.0403 (16)	0.0550 (17)	−0.0087 (14)	0.0236 (14)	−0.0125 (14)
C61	0.0634 (19)	0.0481 (18)	0.0588 (18)	−0.0104 (15)	0.0282 (15)	−0.0106 (15)
C62	0.072 (2)	0.079 (2)	0.0597 (19)	0.0017 (19)	0.0336 (17)	−0.0037 (18)
C63	0.086 (2)	0.087 (3)	0.058 (2)	0.003 (2)	0.0347 (18)	−0.0041 (19)
C64A	0.089 (4)	0.091 (5)	0.063 (4)	0.000 (4)	0.011 (3)	−0.007 (3)
C65A	0.170 (9)	0.137 (8)	0.179 (9)	−0.012 (6)	−0.100 (7)	−0.006 (7)
C66A	0.148 (8)	0.187 (10)	0.172 (9)	0.041 (7)	−0.055 (7)	−0.071 (8)
C67A	0.141 (7)	0.144 (7)	0.095 (6)	0.004 (6)	0.015 (5)	0.049 (6)
C68A	0.115 (6)	0.167 (9)	0.133 (7)	−0.004 (6)	−0.008 (5)	0.063 (6)
C64B	0.106 (10)	0.084 (7)	0.089 (8)	0.009 (7)	−0.001 (9)	0.026 (8)
C65B	0.157 (13)	0.122 (9)	0.094 (10)	0.062 (9)	−0.041 (9)	−0.064 (9)
C66B	0.219 (16)	0.115 (15)	0.164 (17)	−0.077 (13)	−0.138 (14)	0.050 (13)
C67B	0.134 (13)	0.087 (8)	0.044 (7)	0.023 (8)	0.024 (7)	0.010 (6)
C68B	0.26 (3)	0.085 (11)	0.16 (2)	0.034 (16)	0.118 (18)	0.032 (14)
O1	0.0543 (13)	0.130 (2)	0.0708 (15)	−0.0224 (14)	0.0317 (12)	−0.0290 (15)
O2	0.0489 (12)	0.0793 (16)	0.0573 (13)	−0.0139 (11)	0.0058 (10)	−0.0152 (12)
N1	0.0382 (12)	0.0571 (16)	0.0535 (14)	−0.0060 (11)	0.0110 (10)	−0.0024 (12)
C1	0.0437 (15)	0.062 (2)	0.0556 (17)	−0.0055 (14)	0.0144 (13)	−0.0041 (15)
C2	0.0412 (14)	0.0451 (16)	0.0412 (14)	−0.0019 (12)	0.0115 (11)	0.0015 (12)
C3	0.0401 (13)	0.0308 (14)	0.0382 (13)	0.0039 (11)	0.0060 (11)	0.0048 (11)
C4	0.0396 (14)	0.0333 (14)	0.0416 (14)	0.0033 (11)	0.0060 (11)	0.0027 (11)
C5	0.0438 (15)	0.0454 (17)	0.0501 (16)	−0.0001 (13)	0.0047 (13)	−0.0005 (14)
C6	0.0473 (15)	0.0523 (18)	0.0447 (15)	−0.0047 (13)	0.0192 (12)	−0.0038 (13)
C7	0.0480 (15)	0.0479 (17)	0.0349 (13)	−0.0040 (13)	0.0099 (11)	−0.0038 (12)
C8	0.0403 (14)	0.0323 (14)	0.0341 (13)	0.0028 (11)	0.0087 (10)	0.0027 (11)
C9	0.0372 (13)	0.0296 (13)	0.0350 (13)	0.0053 (10)	0.0062 (10)	0.0056 (10)
C10	0.0377 (13)	0.0319 (14)	0.0367 (13)	0.0058 (11)	0.0057 (10)	0.0044 (11)
C11	0.0424 (14)	0.0475 (16)	0.0346 (13)	0.0039 (13)	0.0090 (11)	−0.0009 (12)
C12	0.0423 (14)	0.0426 (16)	0.0388 (14)	0.0018 (12)	0.0017 (11)	−0.0004 (12)
C13	0.0375 (15)	0.076 (2)	0.070 (2)	−0.0088 (15)	0.0140 (14)	−0.0030 (17)
C14	0.0463 (19)	0.108 (3)	0.094 (3)	0.003 (2)	0.0111 (18)	0.017 (2)
C15	0.063 (3)	0.127 (4)	0.163 (5)	0.009 (3)	0.030 (3)	0.066 (4)
C16	0.0540 (19)	0.078 (2)	0.084 (2)	−0.0181 (18)	0.0174 (17)	−0.003 (2)
C17	0.069 (2)	0.089 (3)	0.083 (3)	−0.003 (2)	0.0249 (19)	0.011 (2)
O3	0.0469 (11)	0.0778 (16)	0.0546 (12)	−0.0203 (11)	0.0091 (9)	−0.0026 (11)
O4	0.0716 (14)	0.106 (2)	0.0383 (11)	−0.0311 (14)	0.0130 (10)	−0.0018 (12)
N2	0.0476 (13)	0.0547 (15)	0.0398 (12)	−0.0105 (11)	0.0029 (10)	0.0031 (11)
C18	0.0389 (15)	0.0470 (17)	0.0499 (16)	−0.0023 (13)	0.0057 (12)	0.0055 (13)
C19	0.0370 (13)	0.0351 (14)	0.0410 (14)	0.0034 (11)	0.0060 (11)	0.0060 (11)
C20	0.0371 (13)	0.0314 (14)	0.0395 (13)	0.0041 (11)	0.0084 (11)	0.0088 (11)
C21	0.0442 (14)	0.0434 (16)	0.0380 (13)	−0.0021 (12)	0.0073 (11)	0.0059 (12)
C22	0.0488 (16)	0.0577 (19)	0.0409 (15)	−0.0081 (14)	0.0065 (12)	0.0069 (14)
C23	0.0334 (13)	0.0438 (16)	0.0476 (15)	−0.0002 (12)	0.0088 (11)	0.0053 (12)
C24	0.0377 (14)	0.0470 (16)	0.0396 (14)	0.0014 (12)	0.0113 (11)	0.0030 (12)
C25	0.0329 (13)	0.0339 (14)	0.0407 (13)	0.0042 (11)	0.0103 (11)	0.0061 (11)
C26	0.0329 (12)	0.0312 (14)	0.0388 (13)	0.0051 (10)	0.0067 (10)	0.0071 (11)
C27	0.0354 (13)	0.0336 (14)	0.0409 (14)	0.0037 (11)	0.0089 (11)	0.0064 (11)
C28	0.0411 (15)	0.0574 (19)	0.0448 (15)	−0.0070 (13)	0.0132 (12)	0.0005 (14)
C29	0.0506 (16)	0.064 (2)	0.0379 (14)	−0.0083 (14)	0.0150 (12)	0.0010 (14)
C30	0.0540 (17)	0.066 (2)	0.0433 (16)	−0.0131 (15)	−0.0007 (13)	0.0020 (15)
C31	0.064 (2)	0.084 (3)	0.060 (2)	−0.0042 (19)	−0.0086 (16)	0.0017 (19)
C32	0.110 (3)	0.084 (3)	0.080 (3)	0.016 (3)	−0.010 (2)	−0.005 (2)
C33	0.064 (2)	0.072 (2)	0.0544 (19)	−0.0141 (17)	0.0055 (15)	−0.0042 (17)
C34	0.088 (3)	0.079 (3)	0.066 (2)	−0.009 (2)	0.0259 (19)	−0.0099 (19)
O5	0.0601 (14)	0.118 (2)	0.0986 (19)	0.0159 (15)	0.0362 (14)	0.0347 (17)
O6	0.0661 (14)	0.0791 (17)	0.0674 (15)	0.0113 (13)	0.0090 (11)	0.0141 (13)
N3	0.0519 (14)	0.0584 (17)	0.0712 (17)	0.0066 (13)	0.0142 (13)	0.0075 (14)
C35	0.0567 (18)	0.059 (2)	0.074 (2)	0.0039 (16)	0.0202 (16)	0.0069 (17)
C36	0.0516 (16)	0.0481 (18)	0.0603 (18)	0.0021 (14)	0.0197 (14)	0.0011 (15)
C37	0.0506 (15)	0.0377 (15)	0.0477 (15)	−0.0031 (13)	0.0131 (12)	−0.0050 (12)
C38	0.0515 (16)	0.0443 (17)	0.0527 (16)	−0.0047 (13)	0.0074 (13)	−0.0022 (13)
C39	0.0566 (18)	0.0470 (18)	0.0580 (18)	−0.0019 (14)	0.0069 (14)	−0.0016 (15)
C40	0.0592 (19)	0.062 (2)	0.0615 (19)	0.0061 (16)	0.0276 (15)	0.0055 (16)
C41	0.0605 (18)	0.0585 (19)	0.0433 (15)	0.0037 (15)	0.0173 (13)	0.0036 (14)
C42	0.0522 (16)	0.0388 (15)	0.0421 (14)	−0.0043 (13)	0.0141 (12)	−0.0029 (12)
C43	0.0493 (15)	0.0388 (15)	0.0428 (14)	−0.0057 (12)	0.0129 (12)	−0.0035 (12)
C44	0.0490 (16)	0.0372 (15)	0.0473 (15)	−0.0050 (12)	0.0126 (12)	−0.0033 (12)
C45	0.0576 (18)	0.0569 (19)	0.0442 (15)	−0.0032 (15)	0.0166 (13)	−0.0014 (14)
C46	0.0617 (18)	0.0520 (18)	0.0430 (15)	−0.0020 (15)	0.0094 (13)	0.0013 (14)
C47	0.0539 (19)	0.078 (2)	0.091 (3)	0.0098 (18)	0.0121 (18)	0.014 (2)
C48	0.054 (2)	0.107 (4)	0.140 (4)	0.006 (2)	0.011 (2)	0.008 (3)
C49	0.079 (3)	0.140 (5)	0.180 (5)	−0.006 (3)	0.005 (3)	−0.052 (4)
C50	0.072 (2)	0.098 (3)	0.126 (4)	0.024 (2)	0.036 (2)	0.003 (3)
C51	0.136 (5)	0.121 (4)	0.174 (5)	0.059 (4)	0.062 (4)	0.024 (4)

### Geometric parameters (Å, °)

**Table:**

O7—C52	1.222 (5)	C13—C16	1.527 (5)
O8—C56	1.221 (5)	C14—C15	1.511 (7)
O1—C1	1.212 (4)	C16—C17	1.509 (5)
O2—C5	1.217 (4)	C6—H6	0.9300
O3—C18	1.213 (4)	C7—H7	0.9300
O4—C22	1.220 (3)	C11—H11	0.9300
O5—C35	1.216 (4)	C12—H12	0.9300
O6—C39	1.223 (4)	C13—H13	0.9800
N4—C64B	1.59 (3)	C14—H14B	0.9700
N4—C64A	1.479 (9)	C14—H14A	0.9700
N4—C56	1.394 (5)	C15—H15A	0.9600
N4—C52	1.403 (5)	C15—H15B	0.9600
N1—C1	1.403 (4)	C15—H15C	0.9600
N1—C5	1.395 (4)	C16—H16B	0.9700
N1—C13	1.503 (4)	C16—H16A	0.9700
N2—C18	1.400 (3)	C17—H17C	0.9600
N2—C22	1.393 (4)	C17—H17A	0.9600
N2—C30	1.503 (4)	C17—H17B	0.9600
N3—C39	1.393 (4)	C18—C19	1.478 (4)
N3—C35	1.402 (5)	C19—C20	1.406 (3)
N3—C47	1.490 (4)	C19—C23	1.371 (4)
C52—C53	1.467 (5)	C20—C26	1.421 (3)
C53—C57	1.379 (5)	C20—C21	1.411 (4)
C53—C54	1.406 (5)	C21—C29	1.373 (4)
C54—C60	1.414 (4)	C21—C22	1.484 (4)
C54—C55	1.412 (5)	C23—C24	1.390 (4)
C55—C56	1.469 (5)	C24—C25	1.384 (3)
C55—C63	1.373 (5)	C25—C27^iii^	1.469 (3)
C57—C58	1.385 (5)	C25—C26	1.429 (3)
C58—C59	1.386 (5)	C26—C27	1.426 (3)
C59—C60	1.434 (4)	C27—C28	1.389 (4)
C59—C61^i^	1.452 (4)	C28—C29	1.386 (4)
C60—C61	1.430 (4)	C30—C33	1.521 (5)
C61—C62	1.402 (5)	C30—C31	1.523 (5)
C62—C63	1.379 (5)	C31—C32	1.501 (6)
C64A—C65A	1.403 (14)	C33—C34	1.508 (5)
C64A—C67A	1.540 (13)	C23—H23	0.9300
C64B—C67B	1.55 (2)	C24—H24	0.9300
C64B—C65B	1.38 (2)	C28—H28	0.9300
C65A—C66A	1.371 (14)	C29—H29	0.9300
C65B—C66B	1.526 (19)	C30—H30	0.9800
C67A—C68A	1.411 (13)	C31—H31B	0.9700
C67B—C68B	1.41 (3)	C31—H31A	0.9700
C57—H57	0.9300	C32—H32A	0.9600
C58—H58	0.9300	C32—H32C	0.9600
C62—H62	0.9300	C32—H32B	0.9600
C63—H63	0.9300	C33—H33A	0.9700
C64A—H64A	0.9800	C33—H33B	0.9700
C64B—H64B	0.9800	C34—H34B	0.9600
C65A—H65A	0.9700	C34—H34C	0.9600
C65A—H65B	0.9700	C34—H34A	0.9600
C65B—H65D	0.9700	C35—C36	1.477 (5)
C65B—H65C	0.9700	C36—C37	1.410 (4)
C66A—H66A	0.9600	C36—C40	1.368 (4)
C66A—H66C	0.9600	C37—C38	1.409 (4)
C66A—H66B	0.9600	C37—C43	1.419 (4)
C66B—H66F	0.9600	C38—C39	1.480 (4)
C66B—H66D	0.9600	C38—C46	1.375 (4)
C66B—H66E	0.9600	C40—C41	1.386 (4)
C67A—H67B	0.9700	C41—C42	1.387 (4)
C67A—H67A	0.9700	C42—C43	1.427 (4)
C67B—H67D	0.9700	C42—C44^iv^	1.470 (4)
C67B—H67C	0.9700	C43—C44	1.432 (4)
C68A—H68A	0.9600	C44—C45	1.383 (4)
C68A—H68C	0.9600	C45—C46	1.389 (4)
C68A—H68B	0.9600	C47—C48	1.500 (6)
C68B—H68D	0.9600	C47—C50	1.554 (6)
C68B—H68E	0.9600	C48—C49	1.478 (8)
C68B—H68F	0.9600	C50—C51	1.479 (7)
C1—C2	1.481 (4)	C40—H40	0.9300
C2—C3	1.406 (4)	C41—H41	0.9300
C2—C6	1.374 (4)	C45—H45	0.9300
C3—C9	1.421 (3)	C46—H46	0.9300
C3—C4	1.411 (3)	C47—H47	0.9800
C4—C5	1.482 (4)	C48—H48A	0.9700
C4—C12	1.367 (4)	C48—H48B	0.9700
C6—C7	1.395 (4)	C49—H49A	0.9600
C7—C8	1.384 (4)	C49—H49B	0.9600
C8—C10^ii^	1.470 (3)	C49—H49C	0.9600
C8—C9	1.431 (3)	C50—H50A	0.9700
C9—C10	1.422 (3)	C50—H50B	0.9700
C10—C11	1.389 (3)	C51—H51A	0.9600
C11—C12	1.391 (4)	C51—H51B	0.9600
C13—C14	1.506 (5)	C51—H51C	0.9600
			
Cg1···Cg13^v^	3.6863 (19)	Cg5···Cg9^vi^	3.6604 (16)
Cg2···Cg11	3.7041 (19)	Cg6···Cg10	3.5395 (15)
Cg2···Cg12^v^	3.6485 (18)	Cg6···Cg8^vi^	3.7743 (16)
Cg2···Cg13^v^	3.6165 (18)	Cg6···Cg9	3.7544 (16)
Cg3···Cg13	3.7245 (19)	Cg7···Cg9^vi^	3.5613 (15)
Cg4···Cg13	3.6034 (18)	Cg7···Cg10	3.7518 (14)
Cg4···Cg14^v^	3.7518 (18)		
			
C52—N4—C56	122.8 (3)	C15—C14—H14B	109.00
C52—N4—C64A	115.0 (5)	H14A—C14—H14B	108.00
C52—N4—C64B	127.8 (10)	C13—C14—H14B	109.00
C56—N4—C64A	122.2 (5)	C15—C14—H14A	109.00
C56—N4—C64B	109.3 (10)	C13—C14—H14A	109.00
C1—N1—C13	117.7 (2)	H15B—C15—H15C	110.00
C5—N1—C13	119.2 (2)	C14—C15—H15A	109.00
C1—N1—C5	123.1 (2)	C14—C15—H15B	109.00
C18—N2—C22	123.1 (2)	H15A—C15—H15C	110.00
C18—N2—C30	118.9 (2)	H15A—C15—H15B	109.00
C22—N2—C30	118.1 (2)	C14—C15—H15C	109.00
C35—N3—C47	118.7 (3)	C13—C16—H16B	109.00
C39—N3—C47	118.3 (3)	C13—C16—H16A	109.00
C35—N3—C39	123.0 (3)	C17—C16—H16A	109.00
O7—C52—C53	121.5 (3)	H16A—C16—H16B	108.00
N4—C52—C53	118.0 (3)	C17—C16—H16B	109.00
O7—C52—N4	120.5 (3)	C16—C17—H17C	109.00
C52—C53—C57	119.4 (3)	H17A—C17—H17B	110.00
C54—C53—C57	119.9 (3)	C16—C17—H17B	110.00
C52—C53—C54	120.7 (3)	H17B—C17—H17C	109.00
C55—C54—C60	120.4 (3)	H17A—C17—H17C	109.00
C53—C54—C60	120.3 (3)	C16—C17—H17A	109.00
C53—C54—C55	119.3 (3)	O3—C18—N2	121.0 (2)
C56—C55—C63	120.2 (3)	O3—C18—C19	121.1 (2)
C54—C55—C56	120.9 (3)	N2—C18—C19	117.9 (2)
C54—C55—C63	118.9 (3)	C20—C19—C23	119.7 (2)
O8—C56—N4	120.2 (4)	C18—C19—C23	119.7 (2)
N4—C56—C55	117.9 (3)	C18—C19—C20	120.6 (2)
O8—C56—C55	121.8 (4)	C21—C20—C26	120.3 (2)
C53—C57—C58	120.1 (3)	C19—C20—C21	119.5 (2)
C57—C58—C59	122.6 (3)	C19—C20—C26	120.2 (2)
C58—C59—C61^i^	123.0 (3)	C20—C21—C22	120.6 (2)
C60—C59—C61^i^	119.2 (3)	C20—C21—C29	119.2 (2)
C58—C59—C60	117.9 (3)	C22—C21—C29	120.2 (2)
C59—C60—C61	120.8 (3)	O4—C22—N2	121.1 (3)
C54—C60—C61	119.9 (3)	O4—C22—C21	121.1 (3)
C54—C60—C59	119.3 (3)	N2—C22—C21	117.8 (2)
C59^i^—C61—C60	120.0 (3)	C19—C23—C24	120.7 (2)
C59^i^—C61—C62	122.9 (3)	C23—C24—C25	121.9 (2)
C60—C61—C62	117.1 (3)	C24—C25—C26	118.4 (2)
C61—C62—C63	122.4 (3)	C24—C25—C27^iii^	122.6 (2)
C55—C63—C62	121.3 (3)	C26—C25—C27^iii^	119.0 (2)
C65A—C64A—C67A	131.9 (8)	C25—C26—C27	121.8 (2)
N4—C64A—C65A	116.8 (7)	C20—C26—C25	119.1 (2)
N4—C64A—C67A	111.0 (7)	C20—C26—C27	119.1 (2)
C65B—C64B—C67B	144 (2)	C25^iii^—C27—C26	119.2 (2)
N4—C64B—C67B	102.0 (13)	C26—C27—C28	118.5 (2)
N4—C64B—C65B	104.8 (12)	C25^iii^—C27—C28	122.3 (2)
C64A—C65A—C66A	134.1 (10)	C27—C28—C29	121.7 (3)
C64B—C65B—C66B	122.1 (15)	C21—C29—C28	121.2 (3)
C64A—C67A—C68A	118.4 (9)	C31—C30—C33	113.5 (3)
C64B—C67B—C68B	112.9 (16)	N2—C30—C31	112.6 (2)
C58—C57—H57	120.00	N2—C30—C33	111.3 (2)
C53—C57—H57	120.00	C30—C31—C32	114.8 (3)
C59—C58—H58	119.00	C30—C33—C34	114.1 (3)
C57—C58—H58	119.00	C19—C23—H23	120.00
C63—C62—H62	119.00	C24—C23—H23	120.00
C61—C62—H62	119.00	C25—C24—H24	119.00
C62—C63—H63	119.00	C23—C24—H24	119.00
C55—C63—H63	119.00	C27—C28—H28	119.00
N4—C64A—H64A	92.00	C29—C28—H28	119.00
C67A—C64A—H64A	92.00	C28—C29—H29	119.00
C65A—C64A—H64A	92.00	C21—C29—H29	119.00
C67B—C64B—H64B	100.00	N2—C30—H30	106.00
N4—C64B—H64B	100.00	C31—C30—H30	106.00
C65B—C64B—H64B	100.00	C33—C30—H30	106.00
C66A—C65A—H65A	104.00	C32—C31—H31B	109.00
H65A—C65A—H65B	105.00	H31A—C31—H31B	108.00
C66A—C65A—H65B	104.00	C30—C31—H31B	109.00
C64A—C65A—H65A	104.00	C32—C31—H31A	109.00
C64A—C65A—H65B	104.00	C30—C31—H31A	109.00
H65C—C65B—H65D	107.00	C31—C32—H32A	109.00
C66B—C65B—H65D	107.00	C31—C32—H32B	109.00
C64B—C65B—H65D	107.00	H32B—C32—H32C	110.00
C64B—C65B—H65C	107.00	H32A—C32—H32C	109.00
C66B—C65B—H65C	107.00	C31—C32—H32C	110.00
C65A—C66A—H66B	109.00	H32A—C32—H32B	109.00
C65A—C66A—H66C	109.00	C30—C33—H33A	109.00
C65A—C66A—H66A	110.00	C30—C33—H33B	109.00
H66B—C66A—H66C	109.00	C34—C33—H33A	109.00
H66A—C66A—H66C	109.00	C34—C33—H33B	109.00
H66A—C66A—H66B	110.00	H33A—C33—H33B	108.00
C65B—C66B—H66D	110.00	C33—C34—H34C	109.00
H66E—C66B—H66F	109.00	H34A—C34—H34C	109.00
H66D—C66B—H66E	109.00	H34B—C34—H34C	109.00
H66D—C66B—H66F	110.00	H34A—C34—H34B	109.00
C65B—C66B—H66F	110.00	C33—C34—H34A	109.00
C65B—C66B—H66E	109.00	C33—C34—H34B	110.00
C68A—C67A—H67B	108.00	O5—C35—N3	120.9 (3)
C64A—C67A—H67A	108.00	O5—C35—C36	121.5 (3)
C64A—C67A—H67B	108.00	N3—C35—C36	117.5 (3)
C68A—C67A—H67A	108.00	C35—C36—C37	120.3 (3)
H67A—C67A—H67B	107.00	C35—C36—C40	119.9 (3)
C64B—C67B—H67C	109.00	C37—C36—C40	119.7 (3)
C64B—C67B—H67D	109.00	C36—C37—C38	119.8 (3)
H67C—C67B—H67D	108.00	C36—C37—C43	120.3 (3)
C68B—C67B—H67D	109.00	C38—C37—C43	120.0 (3)
C68B—C67B—H67C	109.00	C37—C38—C39	120.2 (3)
H68A—C68A—H68C	110.00	C37—C38—C46	119.7 (3)
C67A—C68A—H68C	110.00	C39—C38—C46	120.1 (3)
H68B—C68A—H68C	110.00	O6—C39—N3	121.3 (3)
H68A—C68A—H68B	109.00	O6—C39—C38	120.6 (3)
C67A—C68A—H68B	109.00	N3—C39—C38	118.1 (3)
C67A—C68A—H68A	109.00	C36—C40—C41	120.8 (3)
C67B—C68B—H68E	109.00	C40—C41—C42	121.7 (3)
H68E—C68B—H68F	110.00	C41—C42—C43	118.7 (3)
H68D—C68B—H68E	109.00	C41—C42—C44^iv^	122.1 (2)
C67B—C68B—H68F	109.00	C43—C42—C44^iv^	119.1 (2)
C67B—C68B—H68D	109.00	C37—C43—C42	118.7 (2)
H68D—C68B—H68F	109.00	C37—C43—C44	119.4 (2)
O1—C1—N1	121.2 (3)	C42—C43—C44	121.9 (2)
N1—C1—C2	117.5 (2)	C43—C44—C45	118.2 (3)
O1—C1—C2	121.3 (3)	C42^iv^—C44—C43	119.0 (2)
C3—C2—C6	119.2 (2)	C42^iv^—C44—C45	122.9 (3)
C1—C2—C6	119.7 (2)	C44—C45—C46	122.2 (3)
C1—C2—C3	121.0 (2)	C38—C46—C45	120.6 (3)
C4—C3—C9	120.0 (2)	N3—C47—C48	112.5 (3)
C2—C3—C4	119.5 (2)	N3—C47—C50	110.8 (3)
C2—C3—C9	120.6 (2)	C48—C47—C50	115.4 (3)
C3—C4—C12	119.8 (2)	C47—C48—C49	115.6 (4)
C5—C4—C12	119.9 (2)	C47—C50—C51	112.8 (4)
C3—C4—C5	120.4 (2)	C36—C40—H40	120.00
N1—C5—C4	118.2 (2)	C41—C40—H40	120.00
O2—C5—N1	120.8 (3)	C40—C41—H41	119.00
O2—C5—C4	121.1 (3)	C42—C41—H41	119.00
C2—C6—C7	120.9 (3)	C44—C45—H45	119.00
C6—C7—C8	121.9 (2)	C46—C45—H45	119.00
C7—C8—C9	118.3 (2)	C38—C46—H46	120.00
C9—C8—C10^ii^	118.9 (2)	C45—C46—H46	120.00
C7—C8—C10^ii^	122.8 (2)	N3—C47—H47	106.00
C8—C9—C10	121.9 (2)	C48—C47—H47	106.00
C3—C9—C10	119.1 (2)	C50—C47—H47	106.00
C3—C9—C8	119.1 (2)	C47—C48—H48A	108.00
C8^ii^—C10—C9	119.3 (2)	C47—C48—H48B	108.00
C8^ii^—C10—C11	121.9 (2)	C49—C48—H48A	108.00
C9—C10—C11	118.8 (2)	C49—C48—H48B	108.00
C10—C11—C12	121.4 (2)	H48A—C48—H48B	107.00
C4—C12—C11	120.9 (2)	C48—C49—H49A	109.00
C14—C13—C16	114.1 (3)	C48—C49—H49B	109.00
N1—C13—C16	111.2 (3)	C48—C49—H49C	109.00
N1—C13—C14	113.3 (3)	H49A—C49—H49B	110.00
C13—C14—C15	114.7 (4)	H49A—C49—H49C	110.00
C13—C16—C17	114.0 (3)	H49B—C49—H49C	109.00
C7—C6—H6	120.00	C47—C50—H50A	109.00
C2—C6—H6	120.00	C47—C50—H50B	109.00
C8—C7—H7	119.00	C51—C50—H50A	109.00
C6—C7—H7	119.00	C51—C50—H50B	109.00
C10—C11—H11	119.00	H50A—C50—H50B	108.00
C12—C11—H11	119.00	C50—C51—H51A	109.00
C11—C12—H12	119.00	C50—C51—H51B	109.00
C4—C12—H12	120.00	C50—C51—H51C	109.00
N1—C13—H13	106.00	H51A—C51—H51B	109.00
C16—C13—H13	106.00	H51A—C51—H51C	109.00
C14—C13—H13	106.00	H51B—C51—H51C	109.00
			
C56—N4—C52—O7	−173.3 (4)	C12—C4—C5—N1	−173.8 (2)
C56—N4—C52—C53	7.9 (5)	C12—C4—C5—O2	6.1 (4)
C64A—N4—C52—O7	5.1 (6)	C2—C6—C7—C8	2.0 (4)
C64A—N4—C52—C53	−173.8 (4)	C6—C7—C8—C10^ii^	176.6 (3)
C52—N4—C56—O8	176.3 (4)	C6—C7—C8—C9	−1.0 (4)
C52—N4—C56—C55	−4.8 (5)	C10^ii^—C8—C9—C3	−179.1 (2)
C64A—N4—C56—O8	−2.0 (7)	C10^ii^—C8—C9—C10	0.4 (3)
C64A—N4—C56—C55	176.9 (5)	C7—C8—C9—C10	178.1 (2)
C52—N4—C64A—C65A	77.9 (8)	C7—C8—C10^ii^—C11^ii^	1.7 (4)
C52—N4—C64A—C67A	−107.3 (7)	C9—C8—C10^ii^—C11^ii^	179.4 (2)
C56—N4—C64A—C65A	−103.7 (8)	C7—C8—C10^ii^—C9^ii^	−178.0 (2)
C56—N4—C64A—C67A	71.2 (8)	C7—C8—C9—C3	−1.4 (3)
C5—N1—C1—O1	178.7 (3)	C9—C8—C10^ii^—C9^ii^	−0.4 (3)
C5—N1—C1—C2	−2.2 (4)	C3—C9—C10—C11	−1.1 (3)
C13—N1—C1—O1	−1.9 (4)	C3—C9—C10—C8^ii^	179.1 (2)
C13—N1—C1—C2	177.1 (3)	C8—C9—C10—C8^ii^	−0.4 (3)
C1—N1—C5—O2	176.1 (3)	C8—C9—C10—C11	179.4 (2)
C1—N1—C5—C4	−3.9 (4)	C9—C10—C11—C12	−1.3 (4)
C13—N1—C5—O2	−3.2 (4)	C8^ii^—C10—C11—C12	178.4 (2)
C13—N1—C5—C4	176.8 (2)	C10—C11—C12—C4	2.3 (4)
C1—N1—C13—C14	122.8 (3)	N1—C13—C14—C15	−51.9 (4)
C1—N1—C13—C16	−107.1 (3)	C16—C13—C14—C15	179.5 (3)
C5—N1—C13—C14	−57.8 (4)	N1—C13—C16—C17	61.8 (4)
C5—N1—C13—C16	72.3 (3)	C14—C13—C16—C17	−168.5 (3)
C30—N2—C18—O3	7.2 (4)	O3—C18—C19—C20	172.8 (3)
C30—N2—C18—C19	−173.2 (2)	O3—C18—C19—C23	−7.6 (4)
C18—N2—C22—O4	175.1 (3)	N2—C18—C19—C20	−6.8 (4)
C22—N2—C30—C31	−127.0 (3)	N2—C18—C19—C23	172.9 (2)
C22—N2—C30—C33	104.2 (3)	C18—C19—C20—C26	−177.9 (2)
C18—N2—C22—C21	−4.5 (4)	C23—C19—C20—C21	−177.5 (2)
C30—N2—C22—O4	−3.7 (4)	C18—C19—C20—C21	2.2 (4)
C30—N2—C22—C21	176.7 (2)	C23—C19—C20—C26	2.5 (4)
C18—N2—C30—C31	54.1 (3)	C18—C19—C23—C24	179.4 (3)
C18—N2—C30—C33	−74.6 (3)	C20—C19—C23—C24	−1.0 (4)
C22—N2—C18—O3	−171.6 (3)	C26—C20—C21—C22	−178.5 (2)
C22—N2—C18—C19	8.0 (4)	C26—C20—C21—C29	−0.5 (4)
C35—N3—C39—C38	2.0 (5)	C19—C20—C26—C25	−2.6 (3)
C35—N3—C47—C50	−54.2 (4)	C19—C20—C21—C22	1.5 (4)
C39—N3—C47—C48	−104.2 (4)	C19—C20—C21—C29	179.5 (3)
C47—N3—C39—O6	2.3 (5)	C21—C20—C26—C27	−0.5 (3)
C47—N3—C39—C38	−177.0 (3)	C19—C20—C26—C27	179.5 (2)
C35—N3—C47—C48	76.7 (4)	C21—C20—C26—C25	177.4 (2)
C47—N3—C35—C36	168.7 (3)	C20—C21—C22—O4	179.9 (3)
C35—N3—C39—O6	−178.7 (3)	C20—C21—C22—N2	−0.5 (4)
C39—N3—C35—C36	−10.3 (5)	C29—C21—C22—O4	1.9 (5)
C47—N3—C35—O5	−9.3 (5)	C29—C21—C22—N2	−178.5 (3)
C39—N3—C35—O5	171.8 (3)	C20—C21—C29—C28	1.2 (4)
C39—N3—C47—C50	124.9 (3)	C22—C21—C29—C28	179.2 (3)
N4—C52—C53—C54	−5.9 (5)	C19—C23—C24—C25	−0.5 (4)
N4—C52—C53—C57	174.4 (3)	C23—C24—C25—C27^iii^	−178.6 (2)
O7—C52—C53—C57	−4.5 (5)	C23—C24—C25—C26	0.4 (4)
O7—C52—C53—C54	175.3 (3)	C27^iii^—C25—C26—C20	−179.9 (2)
C52—C53—C57—C58	−178.5 (3)	C27^iii^—C25—C26—C27	−2.0 (3)
C52—C53—C54—C60	−179.3 (3)	C24—C25—C26—C27	179.0 (2)
C57—C53—C54—C55	−179.2 (3)	C24—C25—C26—C20	1.2 (3)
C54—C53—C57—C58	1.8 (5)	C24—C25—C27^iii^—C26^iii^	−179.1 (2)
C52—C53—C54—C55	1.0 (5)	C24—C25—C27^iii^—C28^iii^	1.9 (4)
C57—C53—C54—C60	0.5 (5)	C26—C25—C27^iii^—C26^iii^	1.9 (3)
C53—C54—C55—C56	2.1 (5)	C26—C25—C27^iii^—C28^iii^	−177.0 (2)
C53—C54—C60—C61	179.1 (3)	C20—C26—C27—C25^iii^	179.9 (2)
C55—C54—C60—C61	−1.2 (5)	C25—C26—C27—C28	−177.0 (2)
C60—C54—C55—C63	1.5 (5)	C20—C26—C27—C28	0.9 (3)
C53—C54—C55—C63	−178.9 (3)	C25—C26—C27—C25^iii^	2.0 (3)
C55—C54—C60—C59	178.0 (3)	C26—C27—C28—C29	−0.2 (4)
C60—C54—C55—C56	−177.5 (3)	C25^iii^—C27—C28—C29	−179.2 (3)
C53—C54—C60—C59	−1.6 (4)	C27—C28—C29—C21	−0.8 (5)
C56—C55—C63—C62	178.6 (4)	N2—C30—C33—C34	−69.0 (3)
C54—C55—C63—C62	−0.5 (6)	C31—C30—C33—C34	162.8 (3)
C54—C55—C56—N4	−0.4 (5)	C33—C30—C31—C32	177.9 (3)
C63—C55—C56—O8	−0.5 (6)	N2—C30—C31—C32	50.3 (4)
C63—C55—C56—N4	−179.4 (3)	O5—C35—C36—C37	−171.0 (3)
C54—C55—C56—O8	178.5 (4)	O5—C35—C36—C40	7.5 (5)
C53—C57—C58—C59	−3.0 (5)	N3—C35—C36—C37	11.1 (5)
C57—C58—C59—C60	1.7 (5)	N3—C35—C36—C40	−170.5 (3)
C57—C58—C59—C61^i^	−178.5 (3)	C35—C36—C37—C38	−3.5 (4)
C58—C59—C60—C54	0.6 (4)	C35—C36—C37—C43	176.2 (3)
C60—C59—C61^i^—C60^i^	0.0 (4)	C40—C36—C37—C38	178.1 (3)
C58—C59—C60—C61	179.8 (3)	C40—C36—C37—C43	−2.3 (4)
C61^i^—C59—C60—C54	−179.3 (3)	C35—C36—C40—C41	−178.6 (3)
C60—C59—C61^i^—C62^i^	−179.3 (3)	C37—C36—C40—C41	−0.1 (5)
C58—C59—C61^i^—C60^i^	−179.8 (3)	C36—C37—C38—C39	−5.1 (4)
C58—C59—C61^i^—C62^i^	0.9 (5)	C36—C37—C38—C46	177.4 (3)
C61^i^—C59—C60—C61	0.0 (4)	C43—C37—C38—C39	175.2 (3)
C59—C60—C61—C62	−179.3 (3)	C43—C37—C38—C46	−2.3 (4)
C54—C60—C61—C62	−0.1 (4)	C36—C37—C43—C42	2.4 (4)
C59—C60—C61—C59^i^	0.0 (4)	C36—C37—C43—C44	−177.2 (3)
C54—C60—C61—C59^i^	179.3 (3)	C38—C37—C43—C42	−177.9 (3)
C60—C61—C62—C63	1.1 (5)	C38—C37—C43—C44	2.5 (4)
C59^i^—C61—C62—C63	−178.2 (3)	C37—C38—C39—O6	−173.3 (3)
C61—C62—C63—C55	−0.9 (6)	C37—C38—C39—N3	6.0 (4)
C65A—C64A—C67A—C68A	−17.1 (15)	C46—C38—C39—O6	4.2 (5)
C67A—C64A—C65A—C66A	−29.2 (19)	C46—C38—C39—N3	−176.5 (3)
N4—C64A—C67A—C68A	169.1 (7)	C37—C38—C46—C45	0.6 (5)
N4—C64A—C65A—C66A	144.4 (11)	C39—C38—C46—C45	−177.0 (3)
N1—C1—C2—C6	−176.1 (3)	C36—C40—C41—C42	2.3 (5)
O1—C1—C2—C3	−174.9 (3)	C40—C41—C42—C43	−2.1 (4)
O1—C1—C2—C6	3.0 (5)	C40—C41—C42—C44^iv^	177.7 (3)
N1—C1—C2—C3	6.1 (4)	C41—C42—C43—C37	−0.3 (4)
C3—C2—C6—C7	−0.5 (4)	C41—C42—C43—C44	179.3 (3)
C1—C2—C3—C9	175.9 (2)	C44^iv^—C42—C43—C37	179.9 (3)
C1—C2—C3—C4	−3.4 (4)	C44^iv^—C42—C43—C44	−0.5 (4)
C1—C2—C6—C7	−178.4 (3)	C41—C42—C44^iv^—C43^iv^	−179.3 (3)
C6—C2—C3—C4	178.7 (3)	C41—C42—C44^iv^—C45^iv^	−0.4 (5)
C6—C2—C3—C9	−1.9 (4)	C43—C42—C44^iv^—C43^iv^	0.5 (4)
C2—C3—C4—C12	177.6 (3)	C43—C42—C44^iv^—C45^iv^	179.4 (3)
C9—C3—C4—C5	177.7 (2)	C37—C43—C44—C45	−1.0 (4)
C9—C3—C4—C12	−1.8 (4)	C37—C43—C44—C42^iv^	−179.9 (3)
C2—C3—C9—C8	2.9 (3)	C42—C43—C44—C45	179.4 (3)
C2—C3—C9—C10	−176.7 (2)	C42—C43—C44—C42^iv^	0.5 (4)
C4—C3—C9—C10	2.7 (3)	C43—C44—C45—C46	−0.8 (4)
C2—C3—C4—C5	−3.0 (4)	C42^iv^—C44—C45—C46	178.1 (3)
C4—C3—C9—C8	−177.8 (2)	C44—C45—C46—C38	1.0 (5)
C3—C4—C12—C11	−0.7 (4)	N3—C47—C48—C49	68.0 (5)
C3—C4—C5—O2	−173.4 (3)	C50—C47—C48—C49	−163.5 (4)
C3—C4—C5—N1	6.7 (4)	N3—C47—C50—C51	−150.6 (4)
C5—C4—C12—C11	179.9 (3)	C48—C47—C50—C51	80.0 (5)

Symmetry codes: (i) −*x*, −*y*+1, −*z*; (ii) −*x*+1, −*y*+2, −*z*+1; (iii) −*x*+1, −*y*+1, −*z*+1; (iv) −*x*, −*y*+2, −*z*; (v) *x*, *y*−1, *z*;  (vi) *x*, *y*+1, *z*.

### Hydrogen-bond geometry (Å, °)

**Table:**

*D*—H···*A*	*D*—H	H···*A*	*D*···*A*	*D*—H···*A*
C7—H7···O4^vii^	0.93	2.55	3.170 (4)	125
C13—H13···O1	0.98	2.25	2.745 (4)	110
C14—H14B···O2	0.97	2.37	2.930 (4)	116
C15—H15B···O3^vi^	0.96	2.42	3.323 (5)	156
C16—H16B···O2	0.97	2.53	3.097 (4)	117
C30—H30···O4	0.98	2.27	2.746 (4)	109
C31—H31B···O3	0.97	2.34	2.895 (4)	116
C32—H32C···O2	0.96	2.60	3.453 (5)	148
C33—H33A···O3	0.97	2.53	3.107 (4)	118
C41—H41···O8^viii^	0.93	2.49	3.343 (4)	153
C47—H47···O6	0.98	2.26	2.749 (4)	109
C48—H48B···O5	0.97	2.56	3.127 (5)	118
C49—H49C···O7^vi^	0.96	2.56	3.481 (6)	162
C50—H50B···O5	0.97	2.49	2.880 (5)	104
C64A—H64A···O7	0.98	1.95	2.667 (9)	128
C67A—H67A···O8	0.97	2.60	3.139 (11)	116

Symmetry codes: (vii) *x*, −*y*+3/2, *z*+1/2; (vi) *x*, *y*+1, *z*; (viii) *x*, −*y*+3/2, *z*−1/2.

## References

[bb1] Briseno, A. L., Mannsfeld, S. C. B., Reese, C., Hancock, J., Xiong, Y., Jenekhe, S.A., Bao, Z., Xia, Y. (2007). *Nano Lett.* **7**, 2847–2853. 10.1021/nl071495u 17696562

[bb2] Bruker (2003). *SMART* and *SAINT* Bruker AXS Inc., Madison, Wisconsin, USA.

[bb3] Dodabalapur, A. (2006). *Mater. Today*, **9**, 24–30.

[bb4] Farrugia, L. J. (1997). *J. Appl. Cryst.* **30**, 565.

[bb5] Graser, F. & Hädicke, E. (1980). *Justus Liebigs Ann. Chem.* pp. 1994–2001.

[bb6] Graser, F. & Hädicke, E. (1984). *Justus Liebigs Ann. Chem.* pp. 483–494.

[bb7] Guillermet, O., Mossoyan-Deneux, M., Giorgi, M., Glachant, A. & Mossoyan, J. C. (2006). *Thin Solid Films*, **514**, 25–32.

[bb8] Hädicke, E. & Graser, F. (1986*a*). *Acta Cryst.* C**42**, 189–195.

[bb9] Hädicke, E. & Graser, F. (1986*b*). *Acta Cryst.* C**42**, 195–198.

[bb10] Herbst, W. & Hunger, K. (1993). *Ind. Org. Pigm.* **9**, 447-463.

[bb11] Miśkiewicz, P., Mas-Torrent, M., Jung, J., Kotarba, S., Glowacki, I., Gomar-Nadal, E., Amabilino, D. B., Veciana, J., Krause, B., Carbone, D., Rovira, C. & Ulanski, J. (2006). *Chem. Mater.* **18**, 4724–4729.

[bb12] Mizuguchi, J. (2003). *Z. Kristallogr. New Cryst. Struct.* **218**, 131–132.

[bb13] Newman, C. R., Frisbie, D. A., Da Silvo Filho, D. A., Bredas, J. L., Ewback, P. C. & Mann, K. R. (2004). *Chem. Mater.* **16**, 4436–4451.

[bb14] Sheldrick, G. M. (2002). *SADABS* University of Göttingen. Germany.

[bb15] Sheldrick, G. M. (2008). *Acta Cryst.* A**64**, 112–122. 10.1107/S0108767307043930 18156677

[bb16] Spek, A. L. (2009). *Acta Cryst.* D**65**, 148–155. 10.1107/S090744490804362X PMC2631630 19171970

[bb17] Tracz, A., Ulański, J., Pakula, M. & Kryszewski, M. (1981). Pol. Patent 231177.

[bb18] Westrip, S. P. (2010). *J. Appl. Cryst.* **43**, 920–925.

[bb19] Wiatrowski, M., Dobruchowska, E., Maniukiewicz, W., Pietsch, U., Kowalski, J., Szamel, Z. & Ulański, J. (2010). *Thin Solid Films*, **518**, 2266–2270.

[bb20] Wurthner, F. (2004). *Chem. Commun.* **14**, 1564–1579. 10.1039/b401630k 15263926

